# P-726. Rates of Follow-up Testing for Active-Duty Service Members with Syphilis at Joint Base San Antonio

**DOI:** 10.1093/ofid/ofaf695.937

**Published:** 2026-01-11

**Authors:** James J Marsh, Joseph Marcus

**Affiliations:** Brooke Army Medical Center, San Antonio, TX; Brooke Army Medical Center, San Antonio, TX

## Abstract

**Background:**

Current guidelines recommend follow-up testing for syphilis to confirm treatment efficacy. Syphilis rates are higher in the United States military compared to the general population. Despite these higher rates of infection, previous data on military blood donors demonstrated less than one-third received a follow-up rapid plasma reagin (RPR) test within one year after diagnosis. It is unknown if this low follow-up rate exists across different military cohorts.

**Table 1.**
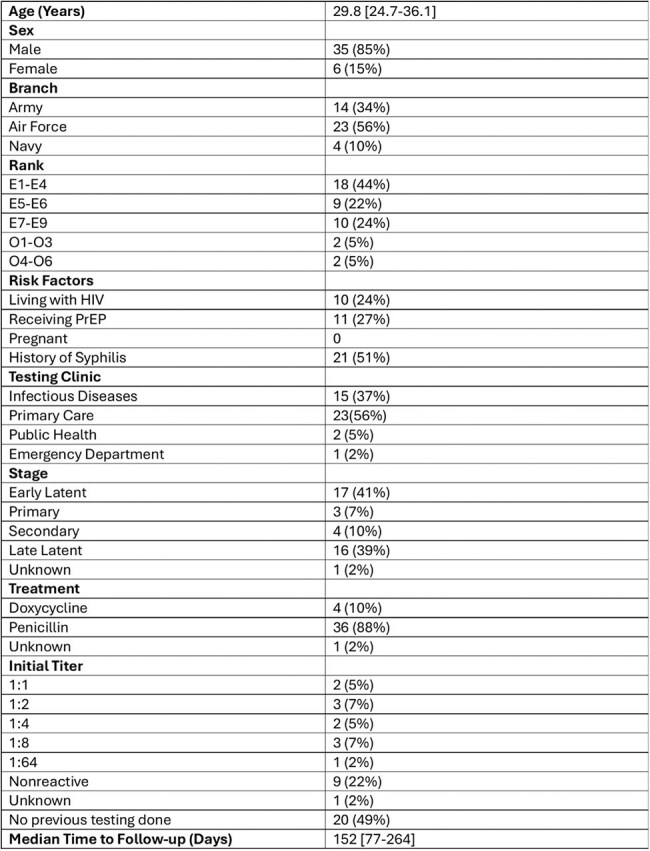
Demographic Information of 41 Individuals Diagnosed with Syphilis at Joint Base San Antonio January 2022-August 2024

**Table 2.**
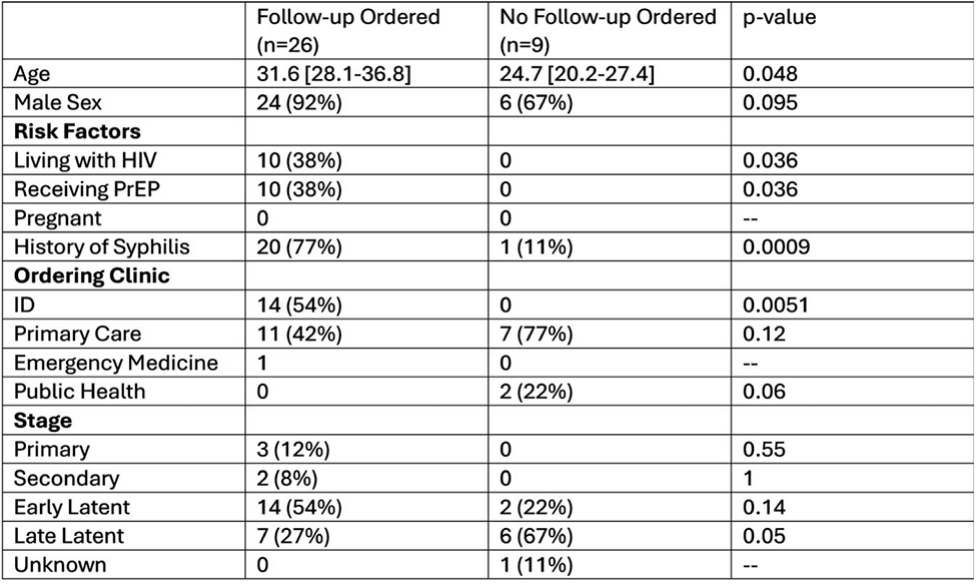
Factors Associated with Follow-up Rapid Plasma Reagin Test After Syphilis Diagnosis

**Methods:**

A retrospective chart review occurred for all active-duty military members with a positive RPR test via reverse algorithm at Joint Base San Antonio between January 2022 and August 2024. Any positive RPR during the study period treated with antimicrobials was considered a true infection. Each chart was reviewed to determine demographic information, testing indication, staging, and follow-up. Patients who received a repeat RPR between 60 days and 365 days were considered to have received a follow-up RPR. A comparison was made between those who received a repeat RPR ordered and those who did not at 365 days. Nominal comparisons were made by Fisher’s Exact Test, and continuous variables were compared by Mann-Whitney U test.

**Results:**

During the study period there were 121 detectable RPR tests in 77 individuals, with 41 (33.8%) determined to be new cases of syphilis. Patients were predominantly male and enlisted. Approximately half were living with HIV or receiving HIV pre-exposure prophylaxis (Table 1). The majority (80%) of cases were staged as latent infections. Of the patients with syphilis, 35 (85%) either had at least one year follow-up after diagnosis or had a repeat RPR ordered (Table 2). The majority (74%) of these individuals had a follow-up RPR ordered within a year. Factors associated with receiving a follow-up RPR included older age, living with HIV, receiving HIV pre-exposure prophylaxis, having a history of syphilis, or receiving testing in the infectious diseases clinic. Patients with late latent syphilis had the lowest follow-up rates.

**Conclusion:**

This study demonstrated a higher rate of follow-up testing than was previously seen in military blood donors. Future interventions should target those with late latent syphilis, as they had the lowest follow-up testing rates.

**Disclosures:**

All Authors: No reported disclosures

